# Optimal domain-specific physical activity and sedentary behaviors for blood lipids among Japanese children: a compositional data analysis

**DOI:** 10.1186/s44167-023-00029-1

**Published:** 2023-10-03

**Authors:** Tetsuhiro Kidokoro, Naruki Kitano, Natsuko Imai, Justin J. Lang, Grant R. Tomkinson, Costan G. Magnussen

**Affiliations:** 1 Faculty of Sport Science, Nippon Sport Science University, 7-1-1, Fukasawa, Setagaya, Tokyo 158-8508 Japan; 2 Alliance for Research in Exercise, Nutrition and Activity (ARENA), University of South Australia, Adelaide, SA Australia; 3 Graduate School of Health and Sports Science, Juntendo University, Chiba, Japan; 4 Physical Fitness Research Institute, Meiji Yasuda Life Foundation of Health and Welfare, Hachioji, Tokyo Japan; 5 Faculty of Child Education and Care, Tokyo Kasei University, Tokyo, Japan; 6 Centre for Surveillance and Applied Research, Public Health Agency of Canada, Ottawa, ON Canada; 7 School of Epidemiology and Public Health, University of Ottawa, Ottawa, ON Canada; 8 Healthy Active Living and Obesity Research Group, Children’s Hospital of Eastern Ontario Research Institute, Ottawa, ON Canada; 9 Baker Heart and Diabetes Institute, Melbourne, VIC Australia; 10 Research Centre of Applied and Preventive Cardiovascular Medicine, University of Turku, Turku, Finland; 11 Centre for Population Health Research, University of Turku and Turku University Hospital, Turku, Finland

**Keywords:** Youth, Cardiometabolic health, School, Time-use epidemiology, Compositional data analysis

## Abstract

**Background:**

Optimizing childhood domain-specific physical activity (PA) and sedentary behavior (SB) for blood lipid profile is not well understood. We aimed to (1) determine the associations between accelerometer-measured PA and SB for each domain (school time and out-of-school time) with blood lipid profile and (2) estimate predicted changes in blood lipid profile with hypothetical time-reallocation between domain-specific SB and PA among Japanese children using compositional data analysis (CoDA).

**Methods:**

This cross-sectional study included 284 children (147 boys and 137 girls) aged 9–12 years (mean age [years]: 10.1 ± 1.2 for boys, 10.0 ± 1.1 for girls; mean body mass index: 18.2 ± 3.2 for boys, 17.5 ± 2.5 for girls). Time spent in domain-specific SB, light-intensity PA (LPA), moderate-intensity PA (MPA), and vigorous-intensity PA (VPA) was assessed using accelerometry. The non-fasting lipid profile considered measures of triglycerides (TG), high-density lipoprotein cholesterol (HDL-C), low-density lipoprotein cholesterol (LDL-C), and non-HDL-C levels. CoDA and isotemporal substitution model were performed to examine the associations of domain-specific PA and SB with blood lipids.

**Results:**

Time spent in out-of-school VPA relative to the other behaviors was negatively associated with non-HDL-C (boys: *β*_ilr1_ = − 0.10, 95% confidence interval [CI] =  − 0.19 to − 0.01), TG (boys: *β*_ilr1_ = − 0.45, 95% CI = − 0.68 to − 0.22), and positively associated with HDL-C (girls: *β*_ilr1_ = 0.09, 95% CI = 0.02 to 0.16) after adjusting for age, body mass index, and time spent in SB, LPA, and MPA. During the out-of-school period, a replacement of 1 min of any other behavior with VPA was associated with decreases in LDL-C in boys (predicted changes [95% CI] − 0.03 mmol/L [− 0.05 to − 0.00] for LPA), non-HDL-C in boys (predicted changes [95% CI] − 0.03 mmol/L [− 0.06 to − 0.01] for SB and LPA) and TG in boys (predicted changes [95% CI] − 0.04 mmol/L [− 0.06 to − 0.02] for SB and LPA, − 0.05 mmol/L [− 0.07 to − 0.02] for MPA) and in girls (predicted changes [95% CI] − 0.02 mmol/L [− 0.04 to − 0.00] for LPA), and increases in HDL-C in girls (predicted changes [95% CI] 0.02 mmol/L [0.00 to 0.04] for SB and LPA, 0.03 mmol/L [0.00 to 0.05] for MPA).

**Conclusions:**

Increasing out-of-school VPA might be an effective approach to improve blood lipid profiles among Japanese children.

**Supplementary Information:**

The online version contains supplementary material available at 10.1186/s44167-023-00029-1.

## Background

Although the associations between daily movement behaviors (i.e., physical activity [PA] [1], sedentary behaviors [SB] [2], and sleep [3]) and health outcomes among children have been studied, these associations have traditionally been examined independently [4]. However, real-world movement behaviors are co-dependent [5], and a change in one movement behavior (e.g., PA) does not occur in isolation [6]. This is because time is finite (24 h per day), and an increase in the time spent on one movement behavior (e.g., PA) is accompanied by a compensatory decrease in others (e.g., SB, sleep, or both) [5, 6]. Therefore, when examining the association between 24-h movement behaviors and health outcomes (e.g., blood lipid profile), it is important to use an analytical approach (e.g., compositional data analysis [CoDA]) that can properly account for the co-dependency of these behaviors [6].

Other studies using CoDA have found inconsistent results when examining the relationship between PA and blood lipids in children [7–10]. For example, Carson et al*.* [7] found a significant positive association between vigorous-intensity PA (VPA) relative to other behaviors (i.e., SB, light-intensity PA [LPA] and moderate-intensity PA [MPA]) and high-density lipoprotein cholesterol (HDL-C) in U.S. children, but not between SB or LPA. In contrast, Hansen et al*.* found that replacing SB with moderate- to vigorous-intensity PA (MVPA) was linked with lower (better) levels of low-density lipoprotein cholesterol (LDL-C) and triglycerides (TG) in Australian children and adolescents [9]. The inconsistency in these results may be explained by domain-specific differences in the optimal reallocation of time between SB and PA [11]. There is a precedence for this in studies of adiposity outcomes. For example, an international study of 4852 adolescents aged 11–19 years found that SB during school time and MVPA during out-of-school time were negatively associated with body mass index (BMI), while out-of-school SB and school-based MVPA were not [12]. A study of Czech children and adolescents aged 8–18 years showed that replacing 30 min/day of out-of-school SB with out-of-school LPA was associated with declines in fat mass by 10% and 14% for boys and girls, respectively; however, no significant associations were found for school SB [13]. Importantly, the reallocation of time between SB and PA are influenced by different factors depending on the domain or subdomain in which the behaviors occur [14]. Because blood lipid levels track strongly from childhood into adulthood [15] and are major risk factors for cardiometabolic disease [16, 17], identifying the domains or subdomains most sensitive to improving blood lipid levels could have important implications for developing and implementing effective interventions. To date, no study has used CoDA to examine the associations between domain-specific movement behaviors and blood lipid profile among children. Using data on Japanese children, the purpose of this study was to (1) examine the associations of school time and out-of-school time PA and SB with blood lipid profile, and (2) estimate predicted changes in blood lipid profile with hypothetical time-reallocation between domain-specific SB and PA using CoDA.

## Methods

### Study design and participants

This cross-sectional study recruited children and adolescents from three public primary schools in Saku City, Nagano, Japan. All children in grades 4 (aged 9–10) and 6 (aged 11–12) attending these schools were invited to participate. All parents/guardians provided written, informed consent on behalf of their children. Data were collected from April to June in 2019 (n = 91) and 2021 (n = 249) and were combined for analysis. Of these, 56 (16.5%) participants were excluded because of incomplete data on blood lipids (n = 10), accelerometry data (n = 41) and sleep duration (n = 25), with the final sample comprising 284 children (147 boys and 137 girls). There were no significant differences in age, sex and anthropometry assessments between participants with incomplete data and final sample. This study was conducted in accordance with the Declaration of Helsinki and approved by the institutional ethical advisory committee of Nippon Sport Science University (project identification code: 021-H005).

## Measurements

### 24-h movement behaviors

PA and SB were measured by three-axis accelerometry (ActiGraphwGT3X-BT, ActiGraph LLC, Pensacola, FL, USA), which exhibits excellent classification accuracy for SB and PA in children [18–20]. Participants were asked to wear the accelerometer on their right hip using a belt for 5 consecutive school days (Monday to Friday), except when sleeping or during water-based activities (e.g., showering, swimming). Data were collected in 15-s epochs. Non-wear time was defined as a period of ≥ 60 min of continuous 0 counts, as recorded by the ActiGraph [21]. Only participants with ≥ 10 h of wear time per day for a minimum of 4 days were included in the analyses [22]. Evenson cut-off points [18] were used to categorize the activities into four levels: SB, < 101 counts per minute (CPM); LPA, 101–2295 CPM; MPA, 2296–4011 CPM; and VPA, ≥ 4012 CPM. Based on the school curriculum provided by the participating schools, domain-specific PA and SB were estimated using the following time segments: (1) school time (i.e., behaviors between 8:20 a.m. and 4:00 p.m.) and (2) out-of-school time (i.e., behaviors outside school time). Accelerometry data were analyzed using ActiLife software (v6.13.3, ActiGraph LLC, Pensacola, FL, USA).

Using a lifestyle questionnaire from the International Study of Childhood Obesity, Lifestyle and the Environment (ISCOLE) study [23], participants were also asked about their bedtime and wake-up time during the previous week on weekdays. Then, the time in bed was calculated from bedtime to wake-up time for each participant. Waking periods were calculated by subtracting the time in bed from 24 h. Subsequently, to standardize the sum of time spent in all movement behavior to 24 h, these waking hours were allocated to SB, LPA, MPA, and VPA in proportion to the total time recorded for each behavior during the accelerometer wear period [24, 25].

### Non-fasting blood lipids

A non-fasting venous blood sample was obtained in the morning by doctors or nurses. Serum total cholesterol (TC) and triglyceride (TG) levels were measured using enzymatic methods, and high-density lipoprotein cholesterol (HDL-C) and low-density lipoprotein cholesterol (LDL-C) were measured using a direct measurement method with an automatic analyzer (LABOSPECT008, Hitachi High-Technologies Corporation, Tokyo, Japan). The non-HDL-C level was calculated by subtracting the HDL-C from TC. In this study, we used HDL-C, LDL-C, non-HDL-C, and TG as blood lipid profile markers and included them in the regression model as a dependent variable.

### Anthropometry assessments

Before anthropometric measurements, the participants were asked to take off their shoes and socks, and heavy clothing (e.g., jacket). Body mass was measured using a digital weighing scale (HD-60B/HD-60P, Nitto Kagaku CO, LTD, Nagoya, Japan) and recorded to the nearest 0.1 kg. Height was measured using a stadiometer (HD-60B/HD-60P, Nitto Kagaku CO, LTD, Nagoya, Japan) and recorded to the nearest 0.1 cm. The body-mass index (BMI) was calculated as body mass (kg) divided by height in meters squared (m^2^).

### Statistical analysis

All analyses were performed with R 4.0.5 (R Foundation for Statistical Computing, Vienna, Austria). Analyses were conducted separately for boys and girls, as the association between PA and SB with blood lipids is expected to differ by sex [26]. Independent t-tests were performed to compare differences in age, movement behavior, body size, and blood lipid profiles between boys and girls.

We used the following two time-use compositions: school time (SB, LPA, MPA, and VPA) and out-of-school time (SB, LPA, MPA, VPA, and time in bed). Time-use compositions were expressed as isometric log-ratios (ilrs) using the pivot coordinate representation [27] (Additional file 1). This resulted in sets of three or four ilr-coordinates from the time-use composition for each domain, where the first one (ilr1) represented time spent in one behavior relative to the remaining behaviors in the domain. For example, the ilr1 coordinate for time-use composition in school time represents the proportion of time spent in SB relative to the other behaviors (LPA, MPA, and VPA). By repeating this procedure, we created ilr1 coordinates for all behaviors in each domain. We report results from ilr1s since our scope is the importance of each PA and SB relative to the remaining parts of time-use composition in each domain. No zero values were recorded for any domain-specific PA or SB.

Compositional multiple linear regression with robust estimators was performed to examine the associations of domain-specific PA and SB with blood lipids. In the regression model, blood lipids and movement behaviors in a specific context (expressed as ilrs) were set as the dependent and independent variables, respectively. Due to skewed distribution, HDL-C, LDL-C, non-HDL-C, and TG were natural log transformed (p values for the Shapiro–Wilk test were < 0.05). Model 1 included age (continuous) and time spent in movement behaviors in the other domain (expressed as ilrs). Model 2 was additionally adjusted for BMI. We selected potential confounders based on previous studies [28] and entered in the model.

We also performed a compositional isotemporal substitution model [29] based on Model 2. In this analysis, the predicted changes in blood lipids were estimated when the time spent in one behavior (e.g., SB in school time) was hypothetically reduced and that spent in another behavior (e.g., VPA in school time) was increased while keeping the other behaviors (e.g., LPA and MPA in school time) constant. Estimates were calculated in 1-min increments up to 10 min. The analysis was only performed for the PA or SB in which statistical significance was observed for the above regression models.

## Results

### Descriptive characteristics

The descriptive characteristics of the study participants are presented in Table 1. During school time, boys spent significantly less time in SB (p < 0.001) and more time in MPA (p < 0.001), and VPA (p < 0.001) than girls. During out-of-school time, boys spent significantly more time in LPA (p = 0.019), MPA (p < 0.001) and VPA (p = 0.005) than girls. In contrast, girls spent significantly more time in SB than boys during out-of-school time (p = 0.001). Time in bed was longer for boys than girls (p = 0.001).

**Table 1:** Descriptive characteristics of the study participants

	Boys (n = 147)		Girls (n = 137)		p value^c^
	Mean^a^	SD/%^b^	Mean^a^	SD/%^b^	
Age (years)	10.1	1.2	10.0	1.1	0.333
Height (cm)	139.1	9.3	137.8	9.2	0.217
Body mass (kg)	35.5	8.9	33.7	8.1	0.067
BMI (kg/m^2^)	18.2	3.2	17.5	2.5	0.057
BMI-z score	0.4	1.6	0.2	1.1	0.089
Blood lipid concentration					
Total cholesterol (mmol/L)	4.4	0.7	4.5	0.7	0.270
Triglyceride (mmol/L)	0.9	0.6	0.9	0.4	0.172
HDL-C (mmol/L)	1.6	0.3	1.6	0.3	0.687
LDL-C (mmol/L)	2.4	0.6	2.5	0.6	0.074
Non-HDL-cholesterol (mmol/L)	2.7	0.6	2.8	0.6	0.137
School time behaviors (min/day)					
Accelerometer wear time	453.3	21.8	451.6	24.7	0.535
SB	256.4	55.6%	301.5	65.4%	< 0.001
LPA	164.0	35.6%	134.9	29.3%	0.340
MPA	27.7	6.0%	17.8	3.9%	< 0.001
VPA	12.9	2.8%	6.9	1.5%	< 0.001
Out-of-school time behaviors (min/day)					
Accelerometer wear time	404.0	115.5	407.6	89.6	0.771
SB	283.8	29.0%	302.1	30.8%	0.001
LPA	129.3	13.2%	120.4	12.3%	0.019
MPA	24.6	2.5%	19.8	2.0%	< 0.001
VPA	7.6	0.8%	5.7	0.6%	0.005
Time in bed	534.7	54.6%	532.0	54.3%	0.001
Meeting PA guideline (%)^d^	63.5		25.0		< 0.001

*SD* standard deviation, *BMI* body mass index, *HDL-C* high-density lipoprotein cholesterol, *LDL-C* low-density lipoprotein cholesterol, *SB* sedentary behaviors, *LPA* light physical activity, *MPA* moderate physical activity, *VPA* vigorous physical activity, *PA* physical activity

^a^Geometric mean normalized to 24-h for time-use components; arithmetic mean for the other variables

^b^The respective geometric means in percentages are presented for 24-h time-use composition

^c^Differences between sexes were tested using the Welch’s t test. For each movement behavior, the first pivot coordinate (i.e., ratio of one behavior and geometric mean of the remaining behaviors) was used to test

^d^Children who spent more than 60 min on MVPA per day met the PA guidelines [38]

### Associations between domain-specific PA and SB with blood lipid profile

**Fig. 1:**
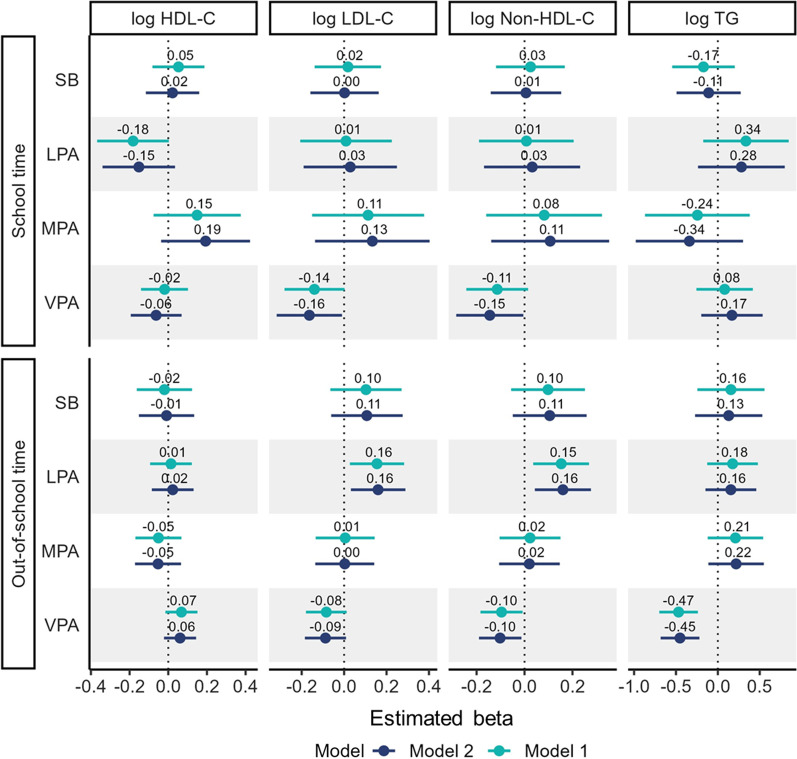
Associations of PA and SB with blood lipid profiles among boys. Model 1 was adjusted for age and PA and SB in the other domains. Model 2 was adjusted for age, PA and SB in the other domains, and body mass index. *SB* sedentary behavior, *LPA* light-intensity physical activity, *MPA* moderate-intensity physical activity, *VPA* vigorous-intensity physical activity, *HDL-C* high-density lipoprotein cholesterol, *LDL-C* low-density lipoprotein cholesterol, *TG* triglyceride. Time-use composition data are expressed as isometric log ratio (ilr) coordinates, and each result was derived from the first set of ilr coordinates, representing the time spent in one behavior relative to the geometric mean of the remaining behaviors in the same domain. Beta coefficients represent the change in the outcome value when time spent in one behavior is increased/decreased, while the geometric mean of the remaining time-use in movement behaviors is accordingly decreased/increased to compensate

Figures 1 (for boys) and 2 (for girls) show the results of the compositional regression analysis between SB, PA, and blood lipids. In boys, significant negative associations were found between out-of-school VPA (relative to the remaining SB and PA behaviors) and non-HDL-C (*β*_ilr1_ = − 0.10, 95% CI = − 0.18 to − 0.01) and TG (*β*_ilr1_ = − 0.47, 95% CI = − 0.70 to − 0.24) (Model 1; Fig. 1 and Additional file 2: Table S1). The associations remained significant for non-HDL-C (*β*_ilr1_ = − 0.10, 95% CI = − 0.19 to − 0.01) and TG (*β*_ilr1_ = − 0.45, 95% CI = − 0.68 to − 0.22) after further adjusting for BMI (model 2). Additionally, there were significant negative associations between school VPA (relative to the remaining SB and PA behaviors) and LDL-C (*β*_ilr1_ = − 0.16, 95% CI = − 0.32 to − 0.01) and non-HDL-C (*β*_ilr1_ = − 0.15, 95% CI = − 0.29 to − 0.01) in Model 2 (Fig. 1 and ﻿Additional file 2: Table S1). In contrast, significant positive associations were found between out-of-school LPA and LDL-C levels (*β*_ilr1_ = 0.16, 95% CI = 0.03 to 0.29) and non-HDL-C levels (*β*_ilr1_ = 0.16, 95% CI = 0.04 to 0.28) in boys (Model 2).

**Fig. 2:**
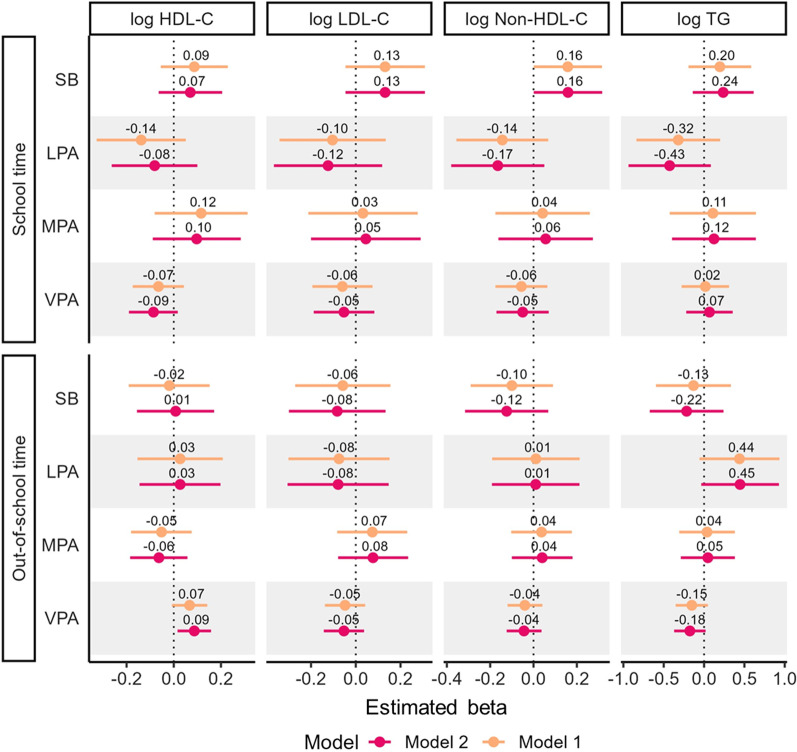
Associations of PA and SB with blood lipid profiles among girls. Model 1 was adjusted for age and PA and SB in the other domains. Model 2 was adjusted for age, PA and SB in the other domains, and body mass index. *SB* sedentary behavior, *LPA* light-intensity physical activity, *MPA* moderate-intensity physical activity, *VPA* vigorous-intensity physical activity, *HDL-C* high-density lipoprotein cholesterol, *LDL-C* low-density lipoprotein cholesterol, *TG* triglyceride. Time-use composition data are expressed as isometric log ratio (ilr) coordinates, and each result was derived from the first set of ilr coordinates, representing the time spent in one behavior relative to the geometric mean of the remaining behaviors in the same domain. Beta coefficients represent the change in the outcome value when time spent in one behavior is increased/decreased, while the geometric mean of the remaining time-use in movement behaviors is accordingly decreased/increased to compensate

For girls, significant positive associations were found between out-of-school VPA and HDL-C (*β*_ilr1_ = 0.09, 95% CI = 0.02 to 0.16) in Model 2 (Fig. 2 and ﻿Additional file 3: Table S2). No significant associations were found between the other PA behaviors and SB and blood lipids in girls.

### Predicted changes in blood lipid profile with hypothetical time-reallocation between domain-specific SB and PA

Our compositional regression analysis confirmed significant associations between VPA and several lipids for both sexes. Therefore, using the compositional isotemporal substitution model, we estimated the predicted change in blood lipids when hypothetically reallocating time from the remaining behaviors to VPA.

**Fig. 3:**
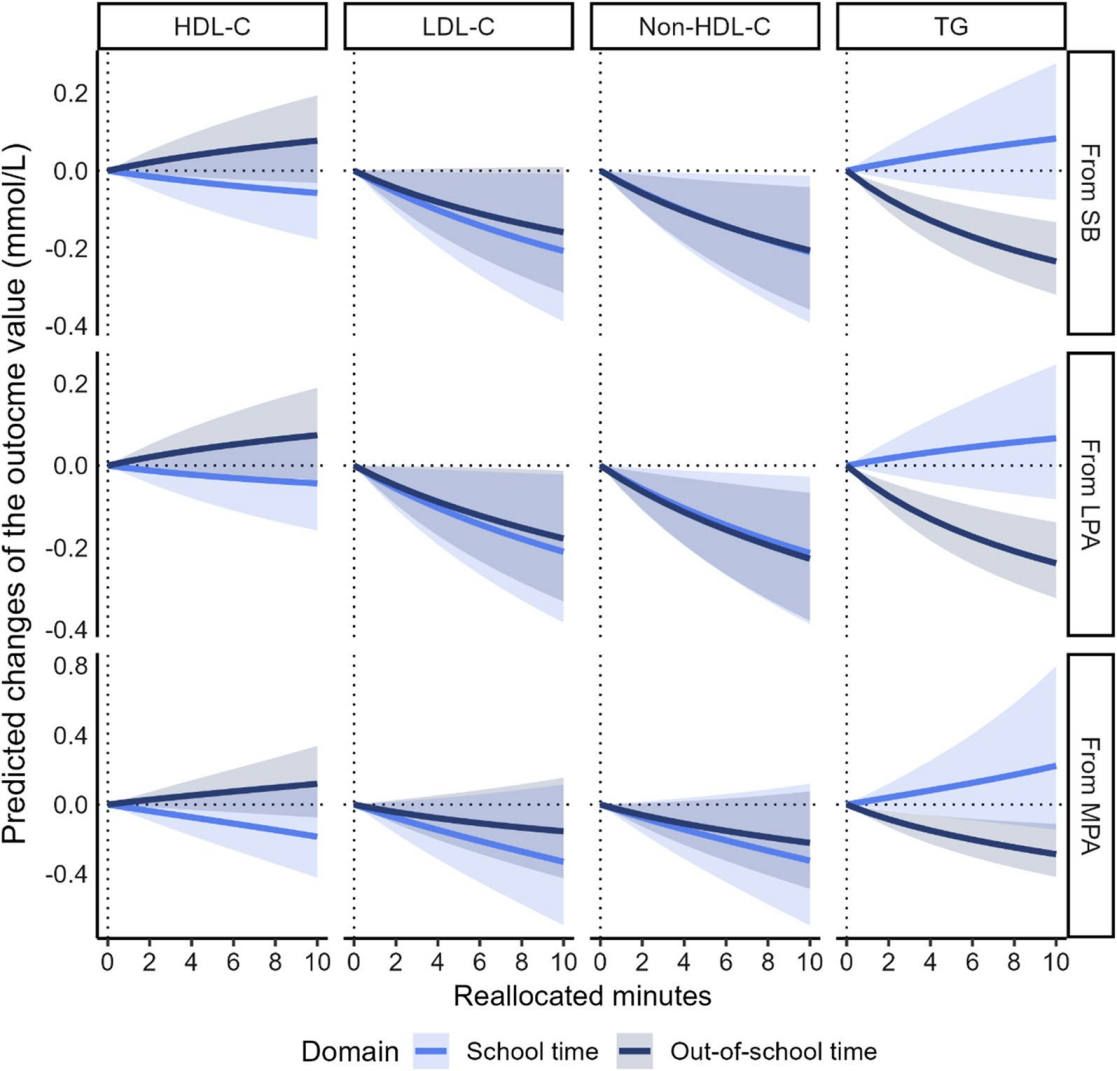
Differences in predicted changes of blood lipid profiles for boys when time spent in one behavior was reduced and VPA was increased with all other behaviors constant. The analysis based on the regression model 2 including following variables as covariates: age, PA and SB in the other domains, and body mass index. *SB* sedentary behavior, *LPA* light-intensity physical activity, *MPA* moderate-intensity physical activity, *VPA* vigorous-intensity physical activity, *HDL-C* high-density lipoprotein cholesterol, *LDL-C* low-density lipoprotein cholesterol, *TG* triglyceride

In boys, only the 1-min time-reallocation from out-of-school SB to out-of-school VPA was associated with lower non-HDL-C levels (− 0.03 mmol/L, 95% CI = − 0.06 to − 0.01) and TG (− 0.04 mmol/L, 95% CI = − 0.06 to − 0.02) (Fig. 3 and ﻿Additional file 4: Table S3). The 1-min time-reallocation from out-of-school LPA to out-of-school VPA was associated with lower LDL-C (− 0.03 mmol/L, 95% CI = − 0.05 to − 0.00), non-HDL-C (− 0.03 mmol/L, 95% CI = − 0.06 to − 0.01), and TG (− 0.04 mmol/L, 95% CI = − 0.06 to − 0.02). The 1-min time-reallocation from out-of-school MPA to out-of-school VPA was associated with lower TG (− 0.05 mmol/L, 95% CI = − 0.07 to − 0.02). Additionally, 1-min time-reallocation from school SB and LPA to school VPA was associated with lower LDL-C (− 0.03 mmol/L, 95% CI = − 0.06 to − 0.00 for both SB and LPA) and non-HDL-C (− 0.03 mmol/L, 95% CI = − 0.06 to − 0.00 for both SB and LPA) (Fig. 3 and ﻿Additional file 4: Table S3).

**Fig. 4:**
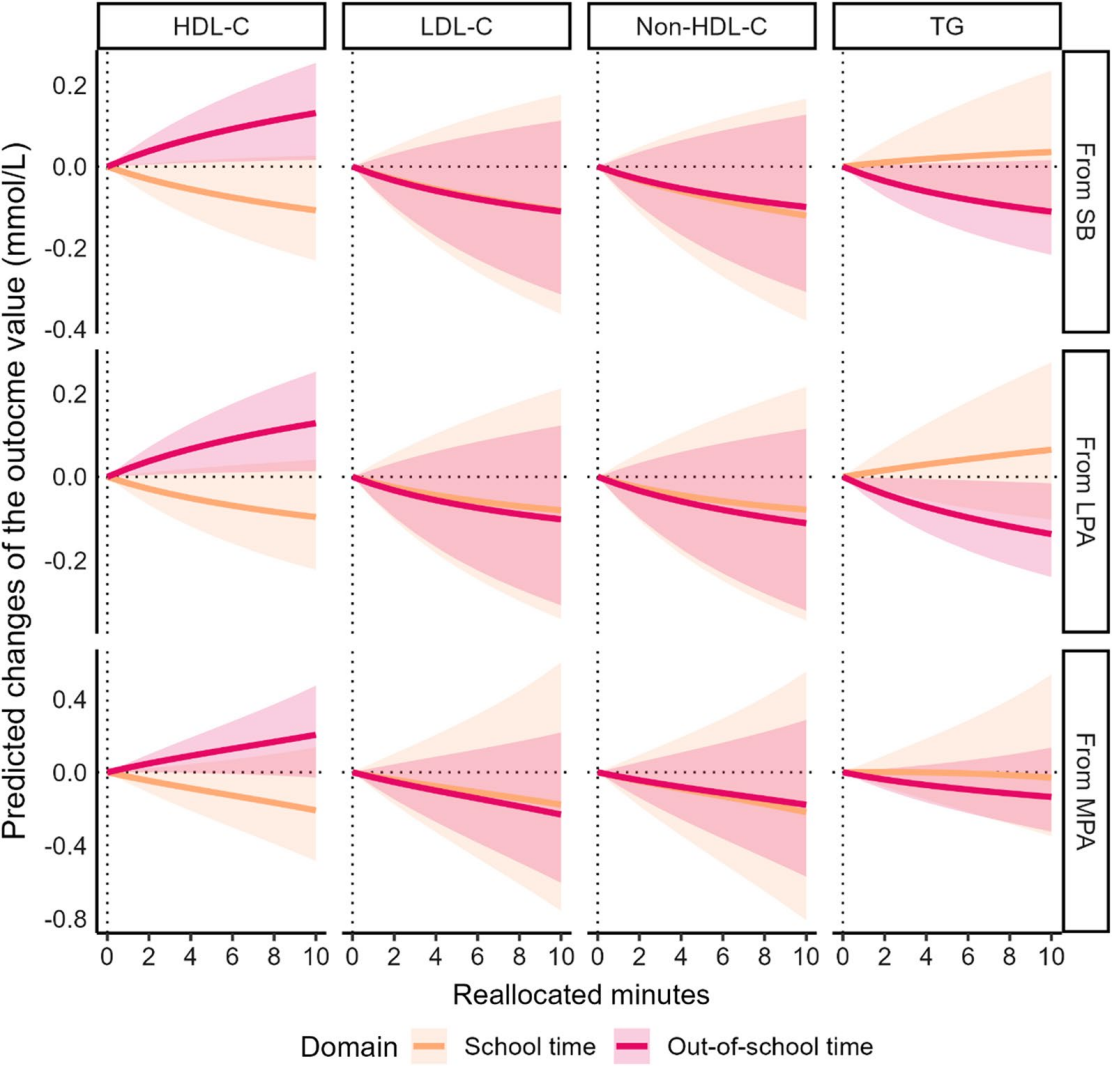
Differences in predicted changes of blood lipid profiles for girls when time spent in one behavior was reduced and VPA was increased with all other behaviors constant. The analysis based on the regression model 2 including following variables as covariates: age, PA and SB in the other domains, and body mass index. *SB* sedentary behavior, *LPA* light-intensity physical activity, *MPA* moderate-intensity physical activity, *VPA* vigorous-intensity physical activity, *HDL-C* high-density lipoprotein cholesterol, *LDL-C* low-density lipoprotein cholesterol, *TG* triglyceride

In girls, the 1-min time-reallocation from any out-of-school behaviors (SB, LPA and MPA) to out-of-school VPA was associated with higher HDL-C (SB and LPA: 0.02 mmol/L, 95% CI = 0.00 to 0.04; MPA: 0.03 mmol/L, 95% CI = 0.00 to 0.05) (Fig. 4 and ﻿Additional file 5: Table S4). Additionally, the 1-min time-reallocation from out-of-school LPA to out-of-school VPA was associated with lower TG (− 0.02 mmol/L, 95% CI = − 0.04 to − 0.00) in girls.

## Discussion

Our findings indicated that spending more time in VPA, particularly outside of school, was associated with better blood lipid profiles. However, we observed weak or uncertain associations for time spent in SB, LPA, and MPA with blood lipids. Using isotemporal substitution analyses, we found that substituting just one minute of SB, LPA, or MPA for VPA was associated with a more favorable blood lipid profile.

### Domain-specific PA and SB for blood lipids

Our findings suggest that out-of-school VPA is particularly important for maintaining blood lipid profile in Japanese children. This finding is consistent with a study of Czech children and adolescents aged 8–18 years [13] that found out-of-school SB, but not school SB, was associated with adiposity. The different associations between school and out-of-school PA and blood lipids might be due to domain-specific differences in PA behaviors. During school time, children typically perform similar PA activities because school environments are relatively more structured [30]. In contrast, out-of-school time tends to be less structured, where children are afforded greater autonomy in PA behaviors, resulting in larger variability among PA behaviors for children during out-of-school time compared to during school time [30]. Additionally, an international comparison study with Japanese and Kenyan children aged 9–12 years showed that Japanese children spent less time in MVPA than Kenyan children, and the greatest differences in MVPA were found during out-of-school period [31]. This suggests that there seems to a room for improvements in out-of-school PA for Japanese children.

### PA intensity and blood lipid profile

We also found that a higher PA intensity might be important for maintaining blood lipid profiles among children, which is consistent with previous findings [12, 32, 33]. Although the beneficial effects of reducing SB and increasing LPA on blood lipids have been well reported in adults [34, 35], the effects are unclear in children [36, 37]. The disagreement between such studies might be due to differences in levels of PA, SB, and blood lipids. Typically, children are more physically active and less sedentary than adults [38], and blood lipid levels for most children fall within the healthy range [28]. For the physically active and metabolically healthy population (i.e., children), reducing SB might not be enough; instead, higher-intensity PA (i.e., VPA) might be required to improve blood lipid levels. It is important to note that although CVD does not typically occur before midlife, the disease process (i.e., atherosclerosis) begins early in life [39]. Given that childhood blood lipid levels track strongly into adulthood [15] and are major risk factors for cardiometabolic disease [16, 17], it is important to maintain healthy blood lipid profiles from an early age.

### Practical and public health implications

Our observation of small increases in VPA (even 1 min/day) associating with better lipid profiles has potential public health implications, particularly given that most children do not meet the recommended PA guideline of an average of 60 min of MVPA per day [40]. While the clinical significance of our findings remains uncertain, the magnitude of blood lipid changes observed seems substantial when compared to previous long-term randomized control trials (RCTs) [41–43]. For example, a 7-year dietary RCT aiming to reduce cholesterol intake among U.S. children with elevated LDL-C levels demonstrated an average reduction of 0.09 mmol/L in the intervention group [41, 42]. Moreover, a RCT involving families with 5-month-old infants who received dietary counselling over 20 years revealed that children in the intervention group exhibited lower average LDL-C levels by 0.10 mmol/L compared to the control group [43]. Our findings suggest that reallocating 1 min of out-of-school LPA to out-of-school VPA associated with a 0.03 mmol/L lower LDL-C, equivalent to 30–33% of the reductions observed in the aforementioned RCTs [41–43]. Considering the current PA landscape among Japanese children, increasing out-of-school VPA by 1 min is a feasible objective. In Japan, active play—such as tag and dodgeball—is a popular out-of-school activity among school-aged children [44], with some of these activities categorized as VPA [45]. Consequently, promoting active play opportunities among Japanese children could potentially improve blood lipid profiles via increased time spent in VPA. This is consistent with the current PA guideline; which recommends that incorporating VPA in addition to 60 min of MVPA [40].

Additionally, we found that VPA, but not MPA, was associated with blood lipids. In most previous studies, MVPA was evaluated as a combination of MPA and VPA, and few studies have separated MPA and VPA in their analyses [7]. A cross-sectional study that considered MPA and VPA separately showed that only VPA was associated with adiposity among 2544 American children aged 6–17 years [7]. Importantly, we found that replacing out-of-school MPA with out-of-school VPA was associated with lower TG levels in boys. This result suggests that increasing activity intensity improves blood lipids without changing the total amount of MVPA, providing an important implication to reimagine current PA practices among children. For example, in Japan, organized sports activities are highly popular, and most Japanese children and adolescents participate in organized sports [44]. These activities are typically performed after school (i.e., out-of-school time) and consist of opportunities to attain VPA [46, 47]. However, a previous study also showed that only 31% of time spent in organized club sport was in MVPA [48]. Given the popularity of organized club sports in Japan and considering children spend most of their play time in LPA during such activities, increasing PA intensity during organized club sport may result in population-level improvements in blood lipids for Japanese children.

### LPA and blood lipid profile results

We found unexpected positive associations between out-of-school LPA and both LDL-C and non-HDL-C levels among boys. While studies have largely focused on the associations between MVPA and blood lipids [1], evidence on the association between LPA and blood lipids has been inconsistent [37, 49]. Future studies should confirm the association between LPA and blood lipids in children.

### Strengths and limitations

This is the first study to examine the associations of domain-specific PA and SB with blood lipids among Japanese children using CoDA, which allowed us to consider the codependency of daily time use. Revealing domain-specific associations of PA behaviors provides important implications for effective intervention strategies for practitioners, educators, and policymakers. However, our study has limitations. First, this study cannot infer causality owing to its cross-sectional design. Second, domain-specific PA (i.e., school versus out-of-school time) was evaluated based on the school curriculum provided by the participating schools; therefore, actual school hours could not be investigated. Although we confirmed that children performed usual practices during data collection, future work should record (e.g., using diaries) when school time starts and ends. Third, we did not include important confounding variables, such as body composition, dietary intake, social economic status, maturation and genetic factors, which are associated with blood lipids [28]. Fourth, only children from three primary schools in Saku City, Nagano Prefecture, were included, which limits the generalizability to the population. Indeed, TC, TG (for girls), and HDL-C levels were significantly lower, and TG levels (for boys) significantly higher, in our sample compared to national norms [50]. Fifth, we did not evaluate weekend PA and SB behaviors, and given the larger variability among PA and SB behaviors during weekend compared to weekday [30], weekend behaviors might be other important factors for blood lipids levels among children.

## Conclusions

We showed a favorable association between the time spent in VPA, particularly out-of-school time, and blood lipid profiles among Japanese children. We found that replacing 1 min of out-of-school SB, LPA, or MPA for VPA was associated with more favorable blood lipid profiles. In contrast, associations for SB, LPA, and MPA were weak or uncertain. These results suggest that increasing out-of-school VPA might be an effective approach for maintaining children’s blood lipid profiles.

## Supplementary Information

**Additional file 1: ** Explanation for isometric log ratio transformation of time-use composition.

**Additional file 2: Table S1.** Associations of physical activity and sedentary behavior with blood lipid profile among boys. Model 1 was adjusted for age and PA and SB in the other domains. Model 2 was adjusted for age, PA and SB in the other domains, and body mass index. SB, sedentary behavior; LPA, light-intensity physical activity; MPA, moderate-intensity physical activity; VPA, vigorous-intensity physical activity; HDL-C, high-density lipoprotein cholesterol; LDL-C, low-density lipoprotein cholesterol; TG, triglyceride. Time-use composition was expressed as isometric log ratio (ilr) coordinates, and each result was from the first ilr coordinates which representing time spent in one behavior relative to the geometric mean of the remaining behaviors in the same domain. Beta coefficients represent the change in the outcome value when time spent in one behavior is increased/decreased, while the geometric mean of the remaining time-use in movement behaviors is accordingly decreased/increased to compensate. Bold values indicate p < 0.05.

**Additional file 3: Table S2.** Associations of physical activity and sedentary behavior with blood lipid profile among girls. Model 1 was adjusted for age and PA and SB in the other domains. Model 2 was adjusted for age, PA and SB in the other domains, and body mass index. SB, sedentary behavior; LPA, light-intensity physical activity; MPA, moderate-intensity physical activity; VPA, vigorous-intensity physical activity; HDL-C, high-density lipoprotein cholesterol; LDL-C, low-density lipoprotein cholesterol; TG, triglyceride. Time-use composition was expressed as isometric log ratio (ilr) coordinates, and each result was from the first ilr coordinates which representing time spent in one behavior relative to the geometric mean of the remaining behaviors in the same domain. Beta coefficients represent the change in the outcome value when time spent in one behavior is increased/decreased, while the geometric mean of the remaining time-use in movement behaviors is accordingly decreased/increased to compensate. Bold values indicate p < 0.05.

**Additional file 4: Table S3.** Differences in predicted changes of blood lipid profile when amounts of time spent in one behavior was reduced and VPA was increased instead while keeping the remaining behaviors constant among boys. The analysis based on the regression model including following variables as covariates: age, PA and SB in the other domains, and body mass index. SB, sedentary behavior; LPA, light-intensity physical activity; MPA, moderate-intensity physical activity; VPA, vigorous-intensity physical activity; HDL-C, high-density lipoprotein cholesterol; LDL-C, low-density lipoprotein cholesterol; TG, triglyceride. Values are in original units before natural log transformation. Bold values indicate p < 0.05.

**Additional file 5: Table S4.** Differences in predicted changes of blood lipid profile when amounts of time spent in one behavior was reduced and VPA was increased instead while keeping the remaining behaviors constant among girls. The analysis based on the regression model including following variables as covariates: age, PA and SB in the other domains, and body mass index. SB, sedentary behavior; LPA, light-intensity physical activity; MPA, moderate-intensity physical activity; VPA, vigorous-intensity physical activity; HDL-C, high-density lipoprotein cholesterol; LDL-C, low-density lipoprotein cholesterol; TG, triglyceride. Values are in original units before natural log transformation. Bold values indicate p < 0.05.

## Data Availability

The datasets used and/or analyzed during the current study are available from the corresponding author on reasonable request.

## Abbreviations

CVD: Cardiovascular disease
PA: Physical activity
SB: Sedentary behavior
MVPA: Moderate-to-vigorous physical activity
LPA: Low-intensity physical activity
BMI: Body mass index
CoDA: Compositional data analysis
CPM: Counts per minute
MPA: Moderate-intensity physical activity
VPA: Vigorous-intensity physical activity
TC: Total cholesterol
TG: Triglyceride
HDL-C: High-density lipoprotein cholesterol
LDL-C: Low-density lipoprotein cholesterol
Ilr: Isometric log ratio
Cis: Confidence intervals

## References

[1] Poitras VJ, Gray CE, Borghese MM, Carson V, Chaput JP, Janssen I, et al. Systematic review of the relationships between objectively measured physical activity and health indicators in school-aged children and youth. Appl Physiol Nutr Metab. 2016;41(6 Suppl 3):S197-239. 27306431 10.1139/apnm-2015-0663

[2] Carson V, Hunter S, Kuzik N, Gray CE, Poitras VJ, Chaput JP, et al. Systematic review of sedentary behaviour and health indicators in school-aged children and youth: an update. Appl Physiol Nutr Metab. 2016;41(6 Suppl 3):S240–65. 27306432 10.1139/apnm-2015-0630

[3] Chaput JP, Gray CE, Poitras VJ, Carson V, Gruber R, Olds T, et al. Systematic review of the relationships between sleep duration and health indicators in school-aged children and youth. Appl Physiol Nutr Metab. 2016;41(6 Suppl 3):S266–82. 27306433 10.1139/apnm-2015-0627

[4] Ferrar K, Chang C, Li M, Olds TS. Adolescent time use clusters: a systematic review. J Adolesc Health. 2013;52(3):259–70. 23299015 10.1016/j.jadohealth.2012.06.015

[5] Pedišić Ž, Dumuid D, Olds TS. Integrating sleep, sedentary behaviour, and physical activity research in the emerging field of time-use epidemiology: definitions, concepts, statistical methods, theoretical framework, and future directions. Kinesiology. 2017;49(2):252–69.

[6] Dumuid D, Pedišić Ž, Palarea-Albaladejo J, Martín-Fernández JA, Hron K, Olds T. Compositional data analysis in time-use epidemiology: what, why, how. Int J Environ Res Public Health. 2020;17(7):2220. 32224966 10.3390/ijerph17072220 PMC7177981

[7] Carson V, Tremblay MS, Chaput JP, McGregor D, Chastin S. Compositional analyses of the associations between sedentary time, different intensities of physical activity, and cardiometabolic biomarkers among children and youth from the United States. PLoS ONE. 2019;14(7): e0220009. 31329609 10.1371/journal.pone.0220009 PMC6645531

[8] Carson V, Tremblay MS, Chaput JP, Chastin SF. Associations between sleep duration, sedentary time, physical activity, and health indicators among Canadian children and youth using compositional analyses. Appl Physiol Nutr Metab. 2016;41(6 Suppl 3):S294-302. 27306435 10.1139/apnm-2016-0026

[9] Hansen BH, Anderssen SA, Andersen LB, Hildebrand M, Kolle E, Steene-Johannessen J, et al. Cross-sectional associations of reallocating time between sedentary and active behaviours on cardiometabolic risk factors in young people: an international children’s accelerometry database (ICAD) analysis. Sports Med. 2018;48(10):2401–12. 29626333 10.1007/s40279-018-0909-1 PMC6132434

[10] Verswijveren S, Lamb KE, Martín-Fernández JA, Winkler E, Leech RM, Timperio A, et al. Using compositional data analysis to explore accumulation of sedentary behavior, physical activity and youth health. J Sport Health Sci. 2022;11(2):234–43. 33737239 10.1016/j.jshs.2021.03.004 PMC9068553

[11] Sallis JF, Owen N, Fisher E. Ecological models of health behavior. In: Health behavior: theory, research, and practice. San Francisco: Jossey-Bass; 2015.

[12] Van Dyck D, Barnett A, Cerin E, Conway TL, Esteban-Cornejo I, Hinckson E, et al. Associations of accelerometer measured school- and non-school based physical activity and sedentary time with body mass index: IPEN adolescent study. Int J Behav Nutr Phys Act. 2022;19(1):85. 35836235 10.1186/s12966-022-01324-x PMC9284738

[13] Gába A, Dygrýn J, Štefelová N, Rubín L, Hron K, Jakubec L. Replacing school and out-of-school sedentary behaviors with physical activity and its associations with adiposity in children and adolescents: a compositional isotemporal substitution analysis. Environ Health Prev Med. 2021;26(1):16. 33504330 10.1186/s12199-021-00932-6 PMC7842010

[14] Klinker CD, Schipperijn J, Toftager M, Kerr J, Troelsen J. When cities move children: development of a new methodology to assess context-specific physical activity behaviour among children and adolescents using accelerometers and GPS. Health Place. 2015;31:90–9. 25463922 10.1016/j.healthplace.2014.11.006

[15] Magnussen CG, Thomson R, Cleland VJ, Ukoumunne OC, Dwyer T, Venn A. Factors affecting the stability of blood lipid and lipoprotein levels from youth to adulthood: evidence from the childhood determinants of adult health study. Arch Pediatr Adolesc Med. 2011;165(1):68–76. 21199983 10.1001/archpediatrics.2010.246

[16] Magnussen CG, Smith KJ, Juonala M. When to prevent cardiovascular disease? As early as possible: lessons from prospective cohorts beginning in childhood. Curr Opin Cardiol. 2013;28(5):561–8. 23928921 10.1097/HCO.0b013e32836428f4

[17] Jacobs DR Jr, Woo JG, Sinaiko AR, Daniels SR, Ikonen J, Juonala M, et al. Childhood cardiovascular risk factors and adult cardiovascular events. N Engl J Med. 2022;386(20):1877–88. 35373933 10.1056/NEJMoa2109191 PMC9563825

[18] Evenson KR, Catellier DJ, Gill K, Ondrak KS, McMurray RG. Calibration of two objective measures of physical activity for children. J Sports Sci. 2008;26(14):1557–65. 18949660 10.1080/02640410802334196

[19] Freedson P, Pober D, Janz KF. Calibration of accelerometer output for children. Med Sci Sports Exerc. 2005;37(11 Suppl):S523–30. 16294115 10.1249/01.mss.0000185658.28284.ba

[20] Trost SG, Loprinzi PD, Moore R, Pfeiffer KA. Comparison of accelerometer cut points for predicting activity intensity in youth. Med Sci Sports Exerc. 2011;43(7):1360–8. 21131873 10.1249/MSS.0b013e318206476e

[21] Masse LC, Fuemmeler BF, Anderson CB, Matthews CE, Trost SG, Catellier DJ, et al. Accelerometer data reduction: a comparison of four reduction algorithms on select outcome variables. Med Sci Sports Exerc. 2005;37(11 Suppl):S544–54. 16294117 10.1249/01.mss.0000185674.09066.8a

[22] Trost SG, Pate RR, Freedson PS, Sallis JF, Taylor WC. Using objective physical activity measures with youth: how many days of monitoring are needed? Med Sci Sports Exerc. 2000;32(2):426–31. 10694127 10.1097/00005768-200002000-00025

[23] Katzmarzyk PT, Barreira TV, Broyles ST, Champagne CM, Chaput JP, Fogelholm M, et al. The international study of childhood obesity, lifestyle and the environment (ISCOLE): design and methods. BMC Public Health. 2013;13:900. 24079373 10.1186/1471-2458-13-900 PMC3849927

[24] McGregor DE, Palarea-Albaladejo J, Dall PM, Hron K, Chastin S. Cox regression survival analysis with compositional covariates: application to modelling mortality risk from 24-h physical activity patterns. Stat Methods Med Res. 2020;29(5):1447–65. 31342855 10.1177/0962280219864125

[25] Del Pozo CB, Alfonso-Rosa RM, McGregor D, Chastin SF, Palarea-Albaladejo J, Del Pozo CJ. Sedentary behaviour is associated with depression symptoms: compositional data analysis from a representative sample of 3233 US adults and older adults assessed with accelerometers. J Affect Disord. 2020;265:59–62. 31959584 10.1016/j.jad.2020.01.023

[26] Martínez-Vizcaíno V, Sánchez-López M, Notario-Pacheco B, Salcedo-Aguilar F, Solera-Martínez M, Franquelo-Morales P, et al. Gender differences on effectiveness of a school-based physical activity intervention for reducing cardiometabolic risk: a cluster randomized trial. Int J Behav Nutr Phys Act. 2014;11:154. 25491026 10.1186/s12966-014-0154-4 PMC4295398

[27] Hron K, Filzmoser P, Thompson K. Linear regression with compositional explanatory variables. J Appl Stat. 2012;39(5):1115–28.

[28] Expert Panel on Integrated Guidelines for Cardiovascular Health and Risk Reduction in Children and Adolescents, National Heart, Lung, and Blood Institute. Expert panel on integrated guidelines for cardiovascular health and risk reduction in children and adolescents: summary report. Pediatrics. 2011;128(Suppl 5):S213–56. 22084329 10.1542/peds.2009-2107C PMC4536582

[29] Dumuid D, Pedišić Ž, Stanford TE, Martín-Fernández JA, Hron K, Maher CA, et al. The compositional isotemporal substitution model: a method for estimating changes in a health outcome for reallocation of time between sleep, physical activity and sedentary behaviour. Stat Methods Med Res. 2019;28(3):846–57. 29157152 10.1177/0962280217737805

[30] Brazendale K, Beets MW, Weaver RG, Pate RR, Turner-McGrievy GM, Kaczynski AT, et al. Understanding differences between summer vs. school obesogenic behaviors of children: the structured days hypothesis. Int J Behav Nutr Phys Act. 2017;14(1):1–14. 28747186 10.1186/s12966-017-0555-2 PMC5530518

[31] Kidokoro T, Fuku N, Yanagiya T, Takeshita T, Takaragawa M, Annear M, et al. Physical activity and sedentary behaviour patterns among Kenyan and Japanese children: a comprehensive cross-country comparison. Int J Environ Res Public Health. 2020;17(12):4254. 32549222 10.3390/ijerph17124254 PMC7344811

[32] García-Hermoso A, Ezzatvar Y, Ramírez-Vélez R, Olloquequi J, Izquierdo M. Is device-measured vigorous physical activity associated with health-related outcomes in children and adolescents? A systematic review and meta-analysis. J Sport Health Sci. 2021;10(3):296–307. 33285309 10.1016/j.jshs.2020.12.001 PMC8167335

[33] Aadland E, Kvalheim OM, Anderssen SA, Resaland GK, Andersen LB. The multivariate physical activity signature associated with metabolic health in children. Int J Behav Nutr Phys Act. 2018;15(1):77. 30111365 10.1186/s12966-018-0707-z PMC6094580

[34] Owen N, Healy GN, Matthews CE, Dunstan DW. Too much sitting: the population health science of sedentary behavior. Exerc Sport Sci Rev. 2010;38(3):105–13. 20577058 10.1097/JES.0b013e3181e373a2 PMC3404815

[35] Füzéki E, Engeroff T, Banzer W. Health benefits of light-intensity physical activity: a systematic review of accelerometer data of the national health and nutrition examination survey (NHANES). Sports Med. 2017;47(9):1769–93. 28393328 10.1007/s40279-017-0724-0

[36] Barnett TA, Kelly AS, Young DR, Perry CK, Pratt CA, Edwards NM, et al. Sedentary behaviors in today’s youth: approaches to the prevention and management of childhood obesity: a scientific statement from the American Heart Association. Circulation. 2018;138(11):e142–59. 30354382 10.1161/CIR.0000000000000591

[37] Väistö J, Haapala EA, Viitasalo A, Schnurr TM, Kilpeläinen TO, Karjalainen P, et al. Longitudinal associations of physical activity and sedentary time with cardiometabolic risk factors in children. Scand J Med Sci Sports. 2019;29(1):113–23. 30276872 10.1111/sms.13315 PMC6485341

[38] Pate RR, O’Neill JR. Summary of the American Heart Association scientific statement: promoting physical activity in children and youth: a leadership role for schools. J Cardiovasc Nurs. 2008;23(1):44–9. 18158507 10.1097/01.JCN.0000305056.96247.bb

[39] Raitakari O, Pahkala K, Magnussen CG. Prevention of atherosclerosis from childhood. Nat Rev Cardiol. 2022;19(8):543–54. 34987194 10.1038/s41569-021-00647-9

[40] Bull FC, Al-Ansari SS, Biddle S, Borodulin K, Buman MP, Cardon G, et al. World Health Organization 2020 guidelines on physical activity and sedentary behaviour. Br J Sports Med. 2020;54(24):1451–62. 33239350 10.1136/bjsports-2020-102955 PMC7719906

[41] Obarzanek E, Kimm SY, Barton BA, Van Horn LL, Kwiterovich PO Jr, Simons-Morton DG, et al. Long-term safety and efficacy of a cholesterol-lowering diet in children with elevated low-density lipoprotein cholesterol: seven-year results of the dietary intervention study in children (DISC). Pediatrics. 2001;107(2):256–64. 11158455 10.1542/peds.107.2.256

[42] Lauer RM, Obarzanek E, Hunsberger SA, Van Horn L, Hartmuller VW, Barton BA, et al. Efficacy and safety of lowering dietary intake of total fat, saturated fat, and cholesterol in children with elevated LDL cholesterol: the dietary intervention study in children. Am J Clin Nutr. 2000;72(5 Suppl):1332s–42s. 11063475 10.1093/ajcn/72.5.1332s

[43] Niinikoski H, Pahkala K, Ala-Korpela M, Viikari J, Rönnemaa T, Lagström H, et al. Effect of repeated dietary counseling on serum lipoproteins from infancy to adulthood. Pediatrics. 2012;129(3):e704–13. 22331346 10.1542/peds.2011-1503

[44] Sasakawa Sports Foundation. The 2021 SSF national sports-life survey of children and young people: Sasakawa Sports Foundation; 2021. https://www.ssf.or.jp/thinktank/survey_list/202106.html. Accessed 11 Apr 2023. (**In Japanese**).

[45] Butte NF, Watson KB, Ridley K, Zakeri IF, McMurray RG, Pfeiffer KA, et al. A Youth compendium of physical activities: activity codes and metabolic intensities. Med Sci Sports Exerc. 2018;50(2):246–56. 28938248 10.1249/MSS.0000000000001430 PMC5768467

[46] Uchiumi K. Extra-curricular school sport (Bukatsu) and corporal punishment in Japan. Asia Pacific J Sport Soc Sci. 2014;3(2):108–14.

[47] Kidokoro T, Tomkinson GR, Lang JJ, Suzuki K. Physical fitness before and during the COVID-19 pandemic: results of annual national physical fitness surveillance among 16,647,699 Japanese children and adolescents between 2013 and 2021. J Sport Health Sci. 2023;12(2):246–54. 36343895 10.1016/j.jshs.2022.11.002 PMC9635948

[48] Sprengeler O, Buck C, Hebestreit A, Wirsik N, Ahrens W. Sports contribute to total moderate to vigorous physical activity in school children. Med Sci Sports Exerc. 2019;51(8):1653–61. 30829902 10.1249/MSS.0000000000001948 PMC6693922

[49] Contardo Ayala AM, Salmon J, Dunstan DW, Arundell L, Timperio A. Does light-intensity physical activity moderate the relationship between sitting time and adiposity markers in adolescents? J Sport Health Sci. 2022;11(5):613–9. 32407803 10.1016/j.jshs.2020.04.002 PMC9532587

[50] Abe Y, Okada T, Sugiura R, Yamauchi K, Murata M. Reference ranges for the non-high-density lipoprotein cholesterol levels in Japanese children and adolescents. J Atheroscler Thromb. 2015;22(7):669–75. 25739922 10.5551/jat.28100

