# Supplementary material for: Optimal domain-specific physical activity and sedentary behaviors for blood lipids among Japanese children: a compositional data analysis
Source: J Act Sedentary Sleep Behav. 2023 Oct 3;2:20. doi: 10.1186/s44167-023-00029-1 (PMC11960305; doi:10.1186/s44167-023-00029-1)
Supplement: Supplementary file 1 — Additional file 1: Explanation for isometric log ratio transformation of time-use composition. [file 44167_2023_29_MOESM1_ESM.docx]

**Additional File 1. Explanation for isometric log ratio transformation of time-use composition.**

Before inclusion in the regression model, two time-use compositions, i.e., school time (sedentary behavior [SB], light-intensity physical activity [LPA], moderate-intensity physical activity [MPA], and vigorous-intensity physical activity [VPA]) and out-of-school time (SB, LPA, MPA, VPA, and time in bed), were expressed as isometric log-ratios (ilrs) using the pivot coordinate representation (Hron et al., 2012). This resulted in sets of three or four ilr-coordinates from the time-use composition for each domain, where the first one (ilr_1_) represented time spent in one behavior relative to that spent in the remaining behaviors in the domain.

$$\begin{aligned} {ilr}_{1}=\sqrt{\frac{3}{4}}ln\frac{{SB}_{school}}{\left( {LPA}_{school}・{MPA}_{school}・{VPA}_{school} \right)^{\frac{1}{3}}}\#\left( 1 \right) \end{aligned}$$

$$\begin{aligned} {ilr}_{2}=\sqrt{\frac{2}{3}}ln\frac{{LPA}_{school}}{\left( {MPA}_{school}・{VPA}_{school} \right)^{\frac{1}{2}}}\#\left( 2 \right) \end{aligned}$$

$$\begin{aligned} {ilr}_{3}=\sqrt{\frac{1}{2}}ln\frac{{MPA}_{school}}{{VPA}_{school}}\#\left( 3 \right) \end{aligned}$$

For example, in the above ilr-coordinates, ilr_1_ represents the ratio of time spent in SB to that spent in LPA, MPA, and VPA during school time. Using time-use composition in school time, we obtained four ilr-coordinate systems by iterating the transformations so that each of the four behaviors in school time was a numerator of the first ilr-coordinate. The same procedure was applied to time-use composition in out-of-school time, so that five coordinate systems, where each of the five behaviors (SB, LPA, MPA, VPA, and time in bed) is ilr_1_, were obtained.

We used each ilr coordinate as an independent variable in the compositional multiple linear regression analysis. For example, when examining the association of time spent in SB relative to that spent in other behaviors in school time with the outcome, we simultaneously entered the above ilr-coordinates, i.e., ilr_1_, ilr_2_, and ilr_3_, into the model.

Reference:

Hron K, Filzmoser P, Thompson K. Linear regression with compositional explanatory variables. J Appl Stat. 2012; 39(5):1115-28.
