# Supplementary material for: Optimal domain-specific physical activity and sedentary behaviors for blood lipids among Japanese children: a compositional data analysis
Source: J Act Sedentary Sleep Behav. 2023 Oct 3;2:20. doi: 10.1186/s44167-023-00029-1 (PMC11960305; doi:10.1186/s44167-023-00029-1)
Supplement: Supplementary file 3 — Additional file 3: Table S2. Associations of physical activity and sedentary behavior with blood lipid profile among girls. Model 1 was adjusted for age and PA and SB in the other domains. Model 2 was adjusted for age, PA and SB in the other domains, and body mass index. SB, sedentary behavior; LPA, light-intensity physical activity; MPA, moderate-intensity physical activity; VPA, vigorous-intensity physical activity; HDL-C, high-density lipoprotein cholesterol; LDL-C, low-density lipoprotein cholesterol; TG, triglyceride. Time-use composition was expressed as isometric log ratio (ilr) coordinates, and each result was from the first ilr coordinates which representing time spent in one behavior relative to the geometric mean of the remaining behaviors in the same domain. Beta coefficients represent the change in the outcome value when time spent in one behavior is increased/decreased, while the geometric mean of the remaining time-use in movement behaviors is accordingly decreased/increased to compensate. Bold values indicate p < 0.05. [file 44167_2023_29_MOESM3_ESM.docx]

Table S2. Associations of physical activity and sedentary behavior with blood lipid profile among girls.

Model 1 was adjusted for age and PA and SB in the other domains. Model 2 was adjusted for age, PA and SB in the other domains, and body mass index. SB, sedentary behavior; LPA, light-intensity physical activity; MPA, moderate-intensity physical activity; VPA, vigorous-intensity physical activity; HDL-C, high-density lipoprotein cholesterol; LDL-C, low-density lipoprotein cholesterol; TG, triglyceride. Time-use composition was expressed as isometric log ratio (ilr) coordinates, and each result was from the first ilr coordinates which representing time spent in one behavior relative to the geometric mean of the remaining behaviors in the same domain. Beta coefficients represent the change in the outcome value when time spent in one behavior is increased/decreased, while the geometric mean of the remaining time-use in movement behaviors is accordingly decreased/increased to compensate. Bold values indicate p<0.05.
