# Supplementary material for: Optimal domain-specific physical activity and sedentary behaviors for blood lipids among Japanese children: a compositional data analysis
Source: J Act Sedentary Sleep Behav. 2023 Oct 3;2:20. doi: 10.1186/s44167-023-00029-1 (PMC11960305; doi:10.1186/s44167-023-00029-1)
Supplement: Supplementary file 5 — Additional file 5: Table S4. Differences in predicted changes of blood lipid profile when amounts of time spent in one behavior was reduced and VPA was increased instead while keeping the remaining behaviors constant among girls. The analysis based on the regression model including following variables as covariates: age, PA and SB in the other domains, and body mass index. SB, sedentary behavior; LPA, light-intensity physical activity; MPA, moderate-intensity physical activity; VPA, vigorous-intensity physical activity; HDL-C, high-density lipoprotein cholesterol; LDL-C, low-density lipoprotein cholesterol; TG, triglyceride. Values are in original units before natural log transformation. Bold values indicate p < 0.05. [file 44167_2023_29_MOESM5_ESM.docx]

Table S4. Differences in predicted changes of blood lipid profile when amounts of time spent in one behavior was reduced and VPA was increased instead while keeping the remaining behaviors constant among girls.

The analysis based on the regression model including following variables as covariates: age, PA and SB in the other domains, and body mass index. SB, sedentary behavior; LPA, light-intensity physical activity; MPA, moderate-intensity physical activity; VPA, vigorous-intensity physical activity; HDL-C, high-density lipoprotein cholesterol; LDL-C, low-density lipoprotein cholesterol; TG, triglyceride. Values are in original units before natural log transformation. Bold values indicate p<0.05.
